# Novel Technique for Noninvasive Detection of Localized Dynamic Brain Signals by Using Transcranial Static Magnetic Fields

**DOI:** 10.1109/JTEHM.2020.3039043

**Published:** 2020-11-18

**Authors:** Osamu Hiwaki

**Affiliations:** Graduate School of Information SciencesHiroshima City University 12801 Hiroshima731-3194Japan

**Keywords:** Biomagnetics, biomedical imaging, encephalography, neural engineering

## Abstract

The techniques for noninvasive measurement of brain function such as electroencephalography (EEG), magnetoencephalography (MEG), functional magnetic resonance imaging (fMRI), and functional near-infrared spectroscopy (fNIRS) have been used in diagnosing brain conditions. However, the conventional techniques have critical limitations of spatial or temporal resolution. Here, we developed a novel technique which enables the precise measurement of dynamic brain signals and localized identification of active brain regions. In this technique, termed as magnetically biased field (MBF), human brain signal is measured as the fluctuation of a transcranial static magnetic field emitted by a coil placed on the scalp. The validity of MBF was confirmed by the measurement of somatosensory evoked signals. Fast somatosensory evoked signals were successfully observed. Localized maximum positive and negative deflections appeared at the region which represents the right primary somatosensory area contralateral to the stimulated hand. The ability of MBF to detect dynamic brain activity precisely can have numerous applications such as diagnosing brain diseases and brain-machine interfaces.

## I. Introduction

Noninvasive methods for the measurement of brain activity have been utilized in diagnosing brain conditions such as seizure, epilepsy, head injuries, dizziness, headache, brain tumors, and sleeping disorders. However, the conventional techniques including electroencephalography (EEG), magnetoencephalography (MEG), functional magnetic resonance imaging (fMRI), and functional near-infrared spectroscopy (fNIRS) have not achieved high resolution in both spatial and temporal domains with one modality. While EEG and MEG can measure fast brain signals in the order of milliseconds, localizing those electromagnetic sources is an ill-posed inverse problem that, in the absence of constraints, does not admit a unique solution because a measured electromagnetic field pattern can be generated by an infinite number of current distributions in the brain [1]–[6]. The signals detected using fMRI and fNIRS originate from fluctuations in the concentration of oxyhemoglobin and deoxy-hemoglobin due to vascular changes in the brain [7]–[14]. The hemodynamic response measured in fMRI and fNIRS is delayed by several seconds following an electrophysiological response. Furthermore, fNIRS features low spatial resolution; the light detector should be placed several tens of mm away from the light source on the head because the emitted light scatters after passing through the cerebral cortex [15]. Approaches to estimation of brain activity with high spatial and temporal resolution by integration of information across different multimodalities, e.g. source localization in EEG/MEG relative to the brain anatomy by the integration with anatomical MRI has been conducted [16]. However, none of conventional noninvasive modalities can alone achieve high resolution in both spatial and temporal domains [17].

To overcome the limitations of the conventional noninvasive methods for measuring brain function, here, we developed magnetically biased field (MBF) as a technique for the precise, localized detection of dynamic brain activity. Further, we verified the validity of MBF by measuring the somatosensory evoked signals in human subjects.

## II. Methods and Procedures

### A. Measuring System of Magnetically Biased Field

**Fig. 1.**
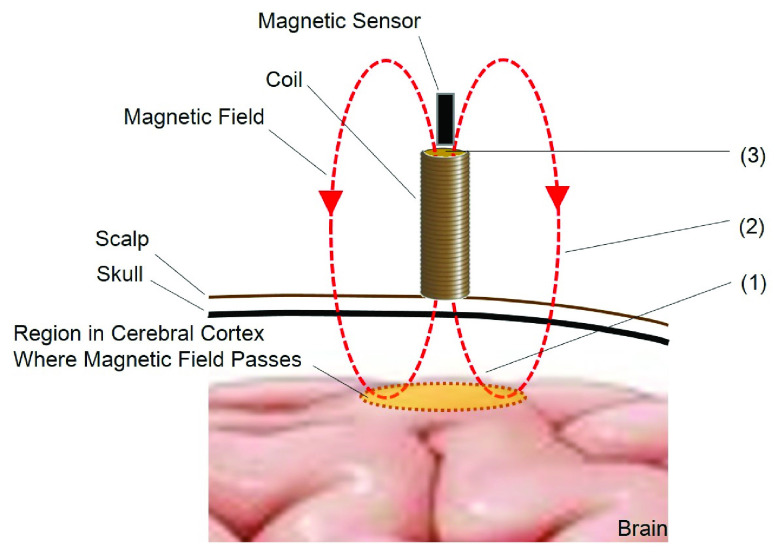
Principle of Magnetically Biased Field (MBF). (1) A static magnetic field generated by a coil on the scalp passes through the cerebral cortex. (2) The static magnetic field fluctuates in accordance with the neural electromagnetic activity in the cerebral cortex through which the static magnetic field passes. (3) The neural activity in the cerebral cortex is measured as a fluctuation of the magnetic field by the magnetic sensor on the top edge of the coil.

In MBF, a static magnetic field is emitted into the head from a coil placed on the scalp, and the magnetic field returned to the coil is measured with a magnetic sensor placed at the top of the coil (Fig. 1). As the emitted static magnetic field fluctuates in accordance with the electromagnetic neural activity it passes through, cortical activity can be measured as the fluctuation of the magnetic field by the magnetic sensor at the top edge of the coil. This technique, therefore, allows for the measurement of localized neural activity at the cortical region where magnetic field passes under the coil.

**Fig. 2.**
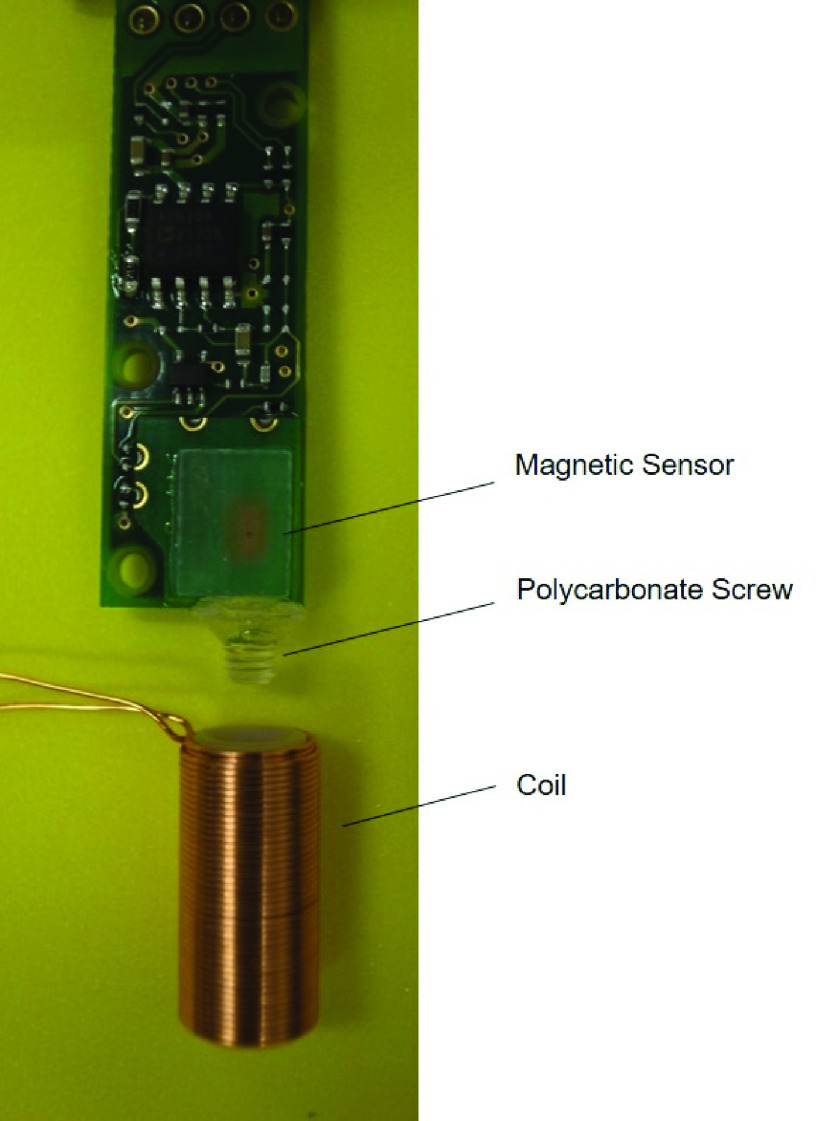
Magnetic sensor and coil for MBF. A magnetic sensor was connected with a coil by a polycarbonate screw rigidly.

To measure human brain signals with MBF, we made a coil producing a static magnetic field; a 28-American wire gauge (AWG, diameter of 0.36 mm) enameled copper wire was wound densely in 2 layers around an acetal resin cylinder with a 6-mm diameter and 15-mm height. The cylinder had a screw-hole situated along the central axis. We adopted a highly sensitive sensor (MI-CB-1DH-M-B, Aichi Steel Corp., Japan) that can measure magnetic field changes at the order of nanotesla in the geomagnetic environment. A polycarbonate screw was attached to the edge of the magnetic sensor along the sensor axis. The magnetic sensor and coil were connected by rigidly tightening the screw into the screw-hole of the coil cylinder (Fig. 2).

**Fig. 3.**
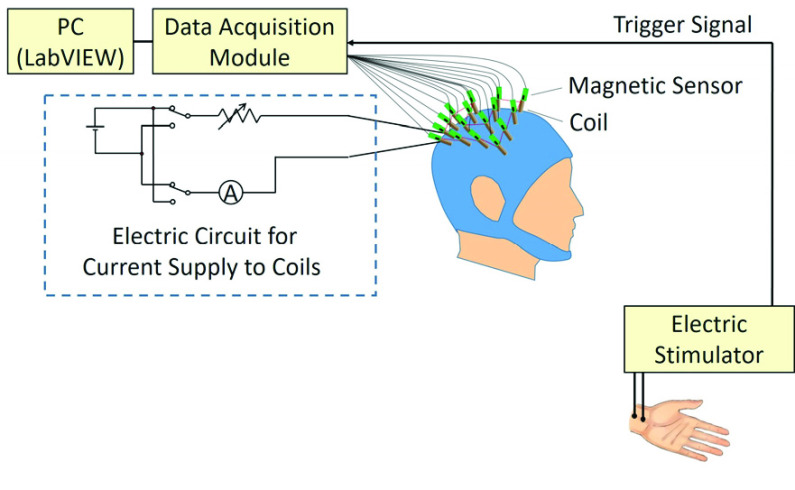
Schematic setup of the experiment. Sixteen sets of magnetic sensors and coils were fixed on a neoprene cap. A direct electric current adjusted by a variable resistance were supplied to the coils. The direction of the electric current was capable to be inverted by a switch. The analog outputs of the magnetic sensors were acquired with a data acquisition module synchronized to the electric stimulation of the median nerve at the left wrist.

We manufactured 16 sets of the magnetic sensors and coils as shown in Fig. 3. The analog outputs of the magnetic sensors were acquired with a data acquisition module (USB-6211, National Instruments Corp., USA). The coils were fixed on a neoprene cap such that each longitudinal axis of the coil and magnetic sensor was situated perpendicular to the scalp. The coils were connected in a series, and a direct electric current was supplied from a 7.2 V nickel-hydrogen battery. The electric current flowing in the coils was adjusted by variable resistance in the electrical circuit. During measurement, we maintained a direct electric current of 100 mA flowing through the coils so as to produce a magnetic field of about $50~\mu \text{T}$, which was nearly as strong as geomagnetic field, at the bottom of each coil cylinder directed in the inward or outward directions of the head.

### B. Measurement of Somatosensory Evoked Signals

**Fig. 4.**
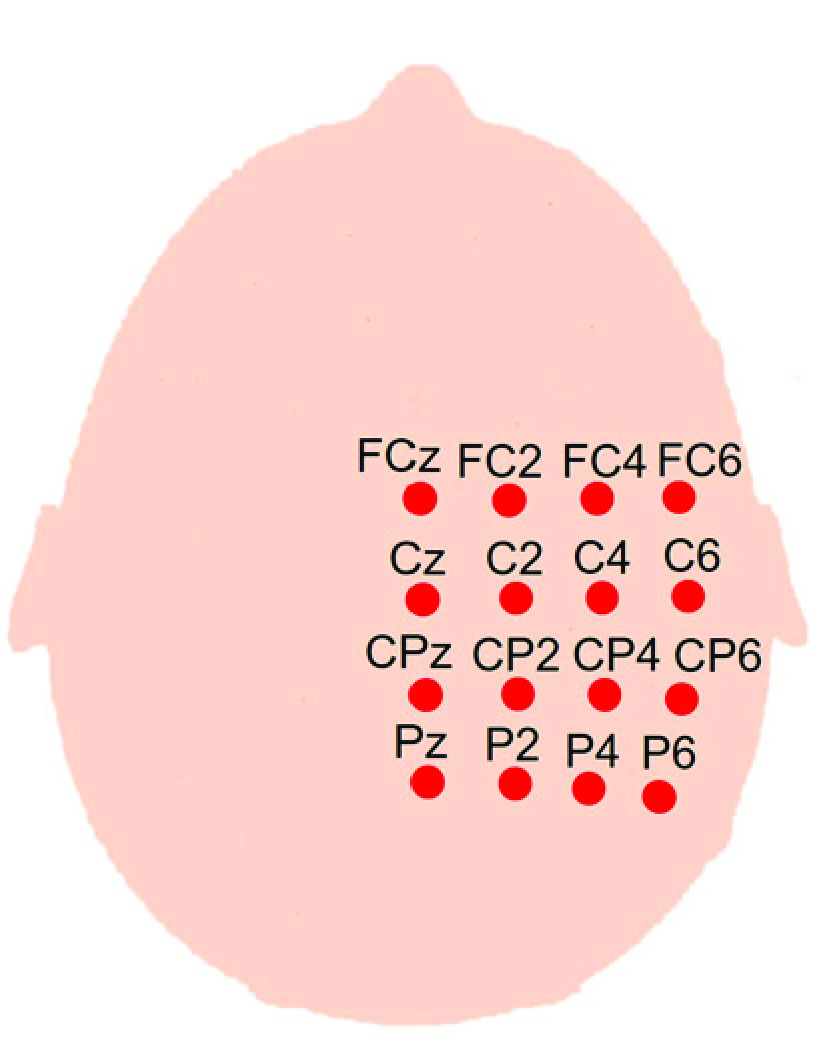
Measurement points located on right hemisphere according to the standard locations of the extended 10-20 EEG recording sites.

We verified the validity of MBF by measuring the somatosensory evoked signals in human subjects. The experiment was performed with five healthy volunteers (2 females and 3 males). The study was approved by the ethical committee of Hiroshima City University, Hiroshima, Japan, and written informed consent was obtained from all subjects. Experiments were conducted in a magnetically shielded room. Median nerve electrical stimulation was applied to the left wrist while the subject sat comfortably in a chair with a mounted headrest. The electric current stimulation consisted of a monophasic square wave pulse with duration of 0.3 ms applied just above the motor threshold to elicit a slight twitch of the thumb, following the recommended standard of International Federation of Clinical Neurophysiology (IFCN) [18]. The target cortical structure aimed was the hand region of S1 primary somatosensory cortex; therefore, we measured the responses at 16 points on right hemisphere localized according to the standard locations of the extended 10-20 EEG recording sites: FCz, FC2, FC4, FC6, Cz, C2, C4, C6, CPz, CP2, CP4, CP6, Pz, P2, P4, and P6 as shown in Fig. 4. The analog signals from the magnetic sensors were digitized at 1 kHz and stored on a personal computer by LabVIEW (National Instruments Corp., USA) from 100 ms before and 500 ms after the stimuli with an interval of 800 ms. The MBF signal was expressed as the fluctuation from the average magnetic field during a 100 ms pre-stimulus period. The somatosensory evoked MBF signals were obtained from an average of 300 responses. To identify the spatial distribution of signal amplitude, topographical mappings were drawn according to a standard MATLAB cubic interpolation routine giving smooth transitions.

## III. Results

**Fig. 5.**
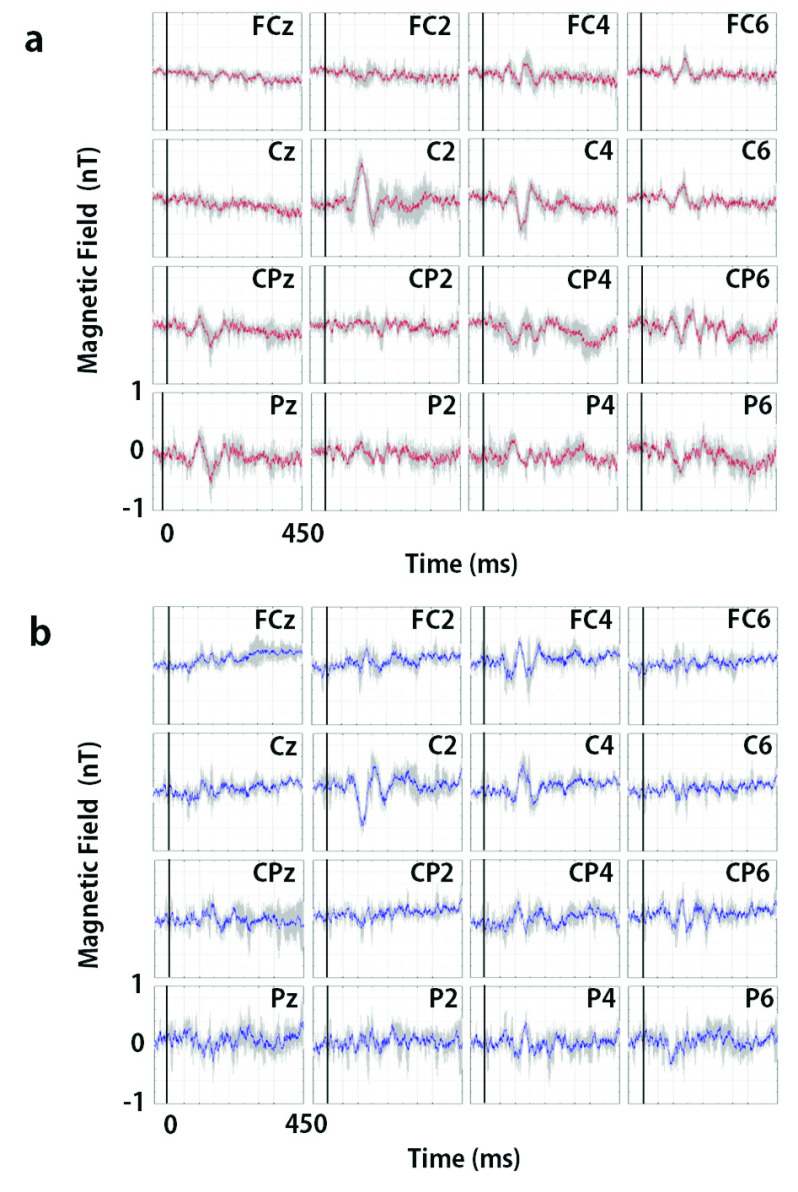
Grand averages of somatosensory evoked MBF signals measured in five subjects. The grey lines indicate the standard deviations at each sampling time. (a) The case of the outward static magnetic field in the coil. (b) The case of the inward static magnetic field in the coil.

**Fig. 6.**
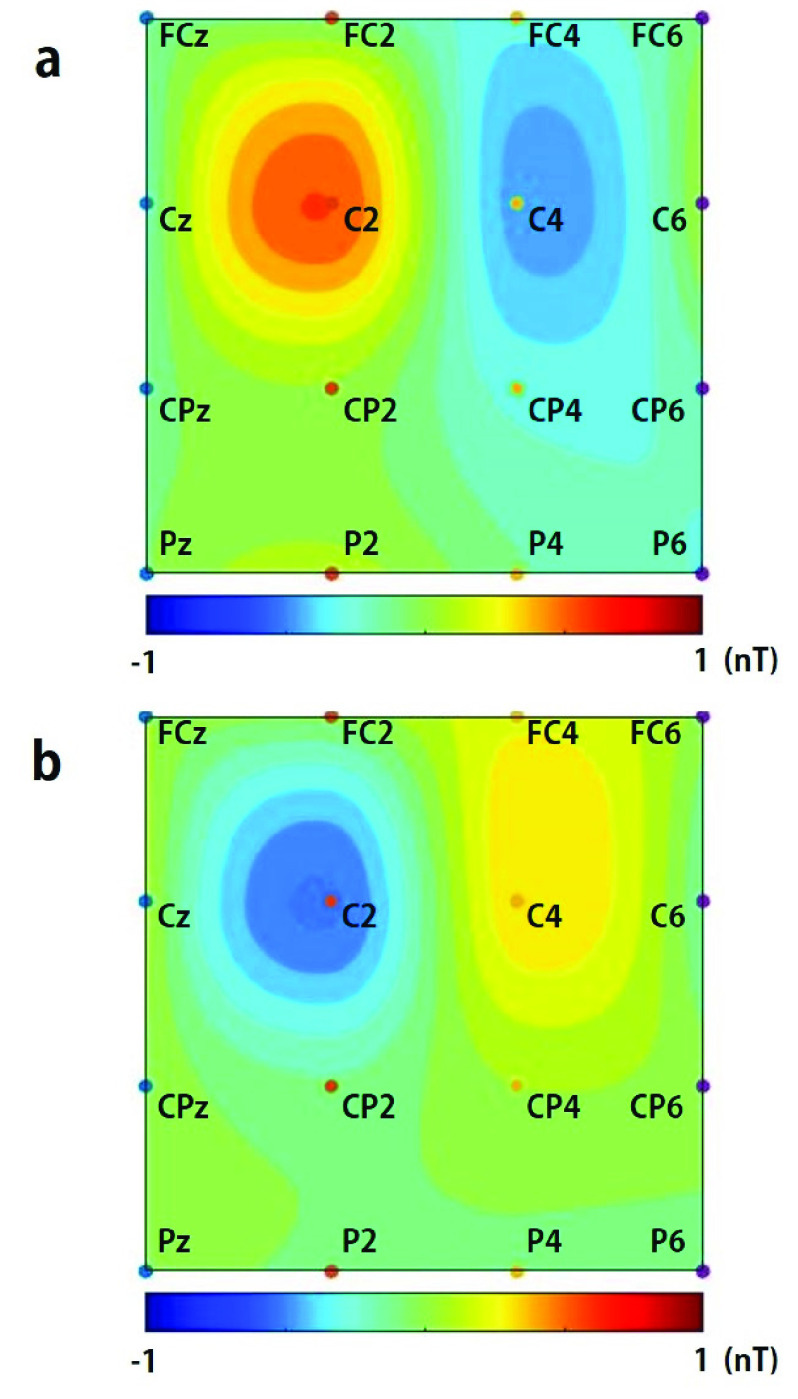
Topographical mappings of MBF signals at the latency of 120 ms. (a) The case of the outward static magnetic field in the coil. (b) The case of the inward static magnetic field in the coil.

Somatosensory evoked MBF signals were thus successfully recorded as shown in Fig. 5. The largest signals were observed at C2 at a latency of 120 ms in both cases of the magnetic field directed in inward and outward direction of the head, whereas little or no signals were observed at FCz, FC2, Cz, CP2, or P2. These findings indicate that the observed signals were not caused by the mechanical movement of the head but rather from brain activity, because the disturbed signals at all measurement points would have appeared if the head movement had influenced. Deviations of the observed signals in the cases of the magnetic field directed in inward and outward direction of the head at C2, C4, FC4, and CP4 were opposite to each other. The signals were so fast that they began to deviate with a latency of 60 ms, and peak latencies were 80 ms, 120 ms, and 160 ms in the C2 signals, which indicates that the signals did not originate from vascular changes that were delayed by several seconds following an electrophysiological response as in fMRI or fNIRS. These peak latencies were not shifted across the subjects. We identified the spatial distribution of MBF signal amplitude by topographical mapping. Fig. 6 illustrates the topographical mapping of the peak latency of 120 ms in the C2 signals in cases of the magnetic field directed in outward (Fig. 6a) and inward (Fig. 6b) direction of the head. The localized maximum positive and negative deflections successfully appeared at latencies of 120 ms at the regions including C2 and C4, which represents the right S1 primary somatosensory area contralateral to the stimulated side. This result shows that the signal distribution representing the primary somatosensory area corresponds to the estimated equivalent current dipole located in the primary sensory cortex by the short latency somatosensory evoked magnetic fields measured with MEG [19], [20]. It should be noted that MBF could detect multiple active regions locally, whereas the estimation of exact source location from MEG data cannot be applied unless a single, not multiple, current dipole can be assumed in the case of short latency somatosensory evoked magnetic fields.

## IV. Discussion

**Fig. 7.**
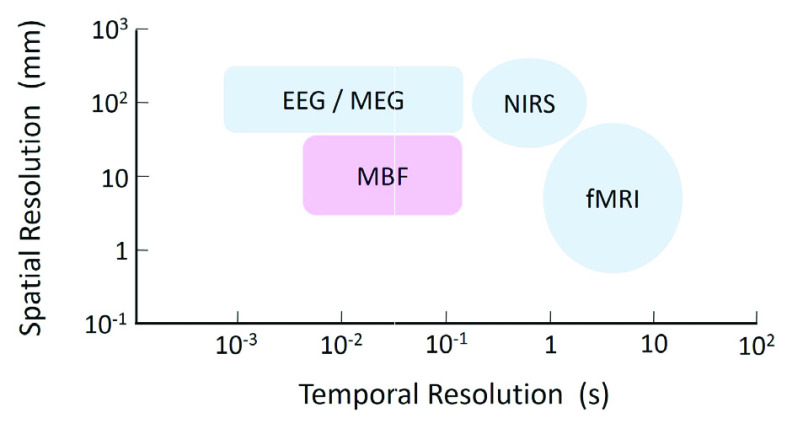
Comparison of spatial and temporal resolution of noninvasive brain imaging techniques. The MBF is considered to be better than the conventional techniques in spatiotemporal resolution.

**Fig. 8.**
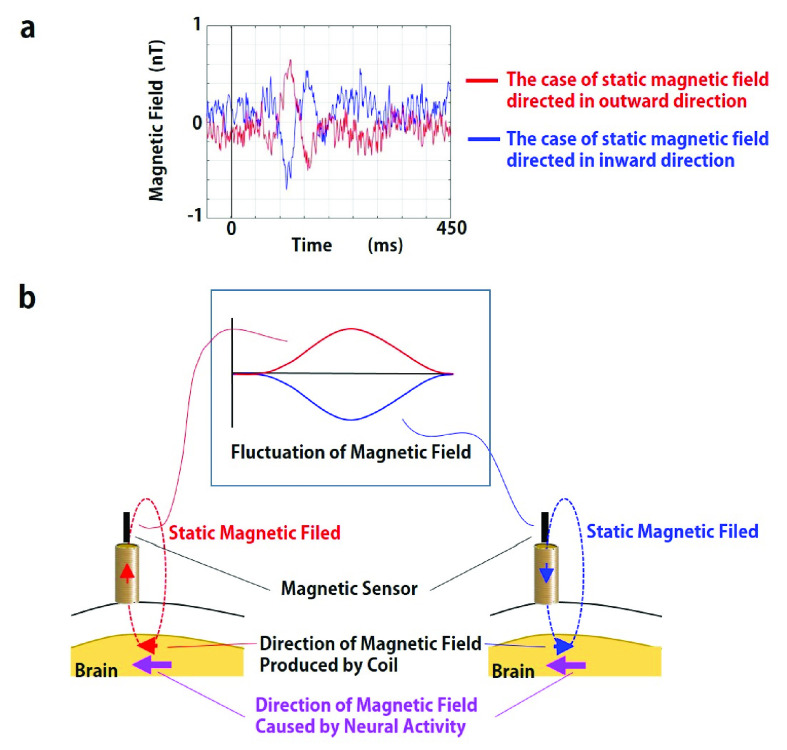
Reversal of the MBF signal depending on the direction of the static magnetic field produced by the coil. (a) Somatosensory evoked MBF signals when the static magnetic fields were directed in the outward and inward direction of the head. Red line: the case of outward directed static magnetic field. Blue line: the case of inward directed static magnetic field. (b) Hypothetical explanation about the signal reversal of the MBF signal. The MBF signal may be reversed depending on the direction of the static magnetic field with respect to the direction of the magnetic field caused by neural activity in the brain.detailed theoretical background underpinning the MBF signal has not been confirmed, MBF signal is thought to be originated from the electrical activity of the brain.

In this study, we achieved the measurement of dynamic brain signals and the localized identification of active brain regions by using transcranial static magnetic fields emitted by coils placed on the scalp, a technique that we call MBF. In the somatosensory evoked MBF signals, main peaks appeared with latencies of about 80 ms, 120 ms, and 160 ms. There have been studies of somatosensory evoked potentials (SEPs) and somatosensory evoked magnetic fields (SEFs) following median nerve stimulation. It is known that large peaks at latencies of about 20 ms and 30 ms in SEPs [21]–[23] and the peaks of similar latencies in SEFs providing the estimated current dipole close to the S1 primary somatosensory cortex [24], [25]. On the other hand, prominent peaks were not observed in the somatosensory evoked MBF responses during the short latency before 60 ms in this study. However, the peaks with latencies of 80 ms, 120 ms, and 160 ms were observed locally at the sites close to the S1 primary somatosensory cortex. The somatosensory evoked potentials measured with 128-channel EEG system could not show such localized mapping indicating the active sites at the S1 primary somatosensory cortex [26]. The overview of the noninvasive neuroimaging methods with respect to their spatial and temporal resolution has been shown as Fig. 7 [19]. It is considered that the property in spatiotemporal resolution of MBF is better than the conventional methods as indicated in Fig. 7. Although the The responses before 200 ms observed in somatosensory evoked MBF are much faster than the signals observed in fMRI and fNIRS caused by the hemodynamic response. It can be discussed, from another point of view, that MBF signal could be an outcome of the electromagnetic neural activity. Deviations of the MBF signals such as at C2 in the cases of the magnetic field directed in inward and outward direction of the head were opposite to each other as shown in Fig. 8a. This phenomenon can be explained with the directional relation between the neural electromagnetic source and the static magnetic field. As shown in the left side of Fig. 8b, when the static magnetic field produced by the coil is directed in same direction of the magnetic field caused by neural activity in the brain, the MBF signal is measured positively by the magnetic sensor. On the other hand, as shown in the right side of Fig. 8b, the static magnetic field in the opposite direction, which is against the direction of the magnetic field caused by the neural activity in the brain, induces negative deflection of the MBF signal. It is not presumable that the reverse deflection of the MBF signal depending on the direction of the static magnetic field is caused by the hemodynamic neural activity which is irrelevant to the electromagnetic property. In EEG and MEG, the signals come from the electrical sources in the brain without any intervention. In contrast, MBF signal is detected in the static magnetic field which is artificially produced. It could be assumed that the larger and delayed response in MBF than those observed in EEG and MEG is attributed to the aggregation of electromagnetic neural sources in the stream of the static magnetic field. The noise level of the magnetic sensor used in this study is 200 pT, which is so low as to detect MBF signal. The MBF signal is so clear that it can be measured without frequency filter in the magnetically shielded room. The large and clear signal provided in MBF is advantageous for measurement of brain function. The MBF measurement can be conducted even in a normal clinical environment if a frequency filter eliminating environmental noise is applied. The MBF measures brain activity with greater efficacy than does fNIRS. The light used in fNIRS, which is emitted radially from the light source situated on the scalp, considerably weakens before returning to the light detector on the scalp because of light scattering [15]. By contrast, following Gauss’s law for magnetism, in the technique of MBF, a static magnetic field emitted from the coil on the scalp that passes through the cerebral cortex returns to the top edge of the coil without scattering as shown in Fig. 1. Due to this property of MBF, the radially directed magnetic neural activity as well as tangentially directed magnetic neural activity could be detected. MEG and fMRI are very costly, not just to purchase but also to maintain, because the expensive liquid helium must be constantly refilled. EEG measurement needs preparation for electrodes with conductive paste or gel to reduce artifact and noise. MBF measurement is much less costly than the MEG and fMRI, and no need for the time-consuming preparation like in EEG. Effects of static magnetic fields on the brain function other than the measurement techniques such as MBF have been reported. Modulation of the excitability of the sensorimotor cortex by applying transcranial static magnetic field stimulation (tSMS) has been demonstrated [27], [28]. The strength of the static magnetic field used in MBF, which is equivalent to the geomagnetic field, is less than 10^−4^ of that in tSMS, so the modulation of the cortex observed in the tSMS is improbable in MBF. This study is the first trial showing the validity of MBF with a measurement of somatosensory evoked signals. It is expected that MBF can be applied to clinical diagnoses by measurements of other functional brain signals such as visual evoked signals, auditory evoked signals and event related signals.

## V. Conclusion

We successfully developed a technique, termed as MBF, for the precise measurement of dynamic brain signals. The ability of MBF to detect dynamic brain activity precisely can aid numerous applications, including cases in which the limitations of conventional noninvasive techniques of brain function render them inadequate in diagnosing brain conditions. MBF can also potentially be applied to brain-machine interfaces (BMI): systems that allow for the direct communication between the brain and external devices [29], [30] because of benefitting from the ease of use, low cost, superior portability, and an ability to measure dynamic brain activity precisely. MBF could open a wide range of possibilities in clinical and general applications.
